# Efficacy and safety of mineralocorticoid receptor antagonists for the treatment of low-renin hypertension: a systematic review and meta-analysis

**DOI:** 10.1038/s41371-023-00891-1

**Published:** 2024-01-11

**Authors:** Sonali S. Shah, Jinghong Zhang, Stella May Gwini, Morag J. Young, Peter J. Fuller, Jun Yang

**Affiliations:** 1 Centre for Endocrinology and Metabolism, Hudson Institute of Medical Research, Clayton, Vic Australia; 2 Department of Endocrinology, Monash Health, Clayton, Vic Australia; 3 Department of Molecular and Translational Science, Monash University, Clayton, Vic Australia

**Keywords:** Adrenal gland diseases, Hypertension

## Abstract

Hypertension is the leading risk factor for premature death. The optimal treatment of low-renin hypertension (LRH), present in 30% of hypertensive individuals, is not known. LRH likely reflects a state of excess salt, expanded volume and/or mineralocorticoid receptor (MR) activation. Therefore, targeted treatment with MR antagonists (MRA) may be beneficial. The objective of this systematic review was to assess the efficacy of MRA therapy in LRH. MEDLINE, Embase and Cochrane databases were searched for randomised controlled trials of adults with LRH that compared the efficacy of MRA to placebo or other antihypertensive treatments. Risk of bias was assessed using the Cochrane risk of bias tool. A meta-analysis was performed using a random-effects model to estimate the difference in blood pressure and the certainty of evidence was assessed using the GRADE approach. The protocol is registered on PROSPERO (CRD42022318763). From the 1612 records identified, 17 studies met the inclusion criteria with a total sample size of 1043 participants. Seven studies (n = 345) were assessed as having a high risk of bias. Meta-analysis indicated that MRA reduced systolic blood pressure by −6.8 mmHg (95% confidence interval −9.6 to −4.1) and −4.8 mmHg (95% confidence interval −11.9 to 2.4) compared to angiotensin-converting enzyme inhibitors/angiotensin receptor blockers (ACEi/ARB) and diuretics. The certainty of the evidence was assessed as moderate and very low, respectively. The findings of this systematic review suggest that MRA is effective in lowering blood pressure in LRH and may be better than ACEi/ARB. Translation to clinical practice is limited by the uncertainty of evidence.

## Introduction

Hypertension is defined as systolic blood pressure (SBP) ≥ 130 mmHg or diastolic blood pressure (DBP) ≥ 80 mmHg [1]. It affects an estimated 1.28 billion adults worldwide and is a major cause of premature death [2]. Adequate control of this modifiable risk factor is key to reducing cardiovascular disease; a 5 mmHg reduction in SBP is associated with a 10% reduction in major cardiovascular events [3]. However, four out of five hypertensive people do not meet blood pressure (BP) targets and it is estimated that two-thirds of patients will require more than one drug to achieve BP control [2].

One possible reason for this is that the current one-size-fits-all sequential approach to pharmacotherapy fails to address the underlying pathophysiology of hypertension for the individual [1, 4]. The renin-angiotensin-aldosterone system is a key regulator of BP. Low dietary salt and blood volume stimulate the release of renin, which leads to a cascade of downstream effects including water and salt reabsorption and increased vascular tone mediated by angiotensin II, aldosterone and mineralocorticoid receptor (MR) activation. However, when the renin-angiotensin-aldosterone system is dysregulated, such as in the case of primary aldosteronism (PA), there is a loss of the negative feedback mechanism, leading to inappropriate MR activation promoting excess sodium and water reabsorption, hypertension and end-organ damage [5]. PA is diagnosed by the presence of low renin and an inappropriately normal or elevated plasma aldosterone concentration, resulting in an elevated aldosterone-to-renin ratio (ARR) [6]. PA has clear targeted treatment options including medical therapy with MR antagonist (MRA) or adrenalectomy in the case of an aldosterone-producing adenoma. Importantly, adrenalectomy to remove the source of excess aldosterone, and MR blockade are effective in reducing BP and the elevated cardiovascular risk associated with PA [7]. With easier access to renin and aldosterone measurement and advocacy for expanded screening for PA, clinicians are faced with the dilemma of how to manage patients who have low renin but do not meet the diagnostic criteria for PA. This condition, known as low-renin hypertension (LRH), is present in as many as 30% of hypertensive individuals [8]. It is hypothesised that the low renin in the context of hypertension reflects excess MR activation and/or salt reabsorption due to abnormalities in renal sodium handling in the distal nephrons of the kidneys [9, 10]. Current clinical practice guidelines do not provide clear recommendations for the initial choice of monotherapy for people with LRH [1, 4]. In a randomised controlled trial (RCT) of participants with resistant hypertension on three antihypertensives, a lower baseline renin was associated with a greater BP-lowering response with MRA add-on therapy compared to a beta-blocker and an alpha-blocker [11]. This raises the question of whether early targeted MRA treatment in patients with LRH would be beneficial and possibly avoid the need for multiple anti-hypertensives. The findings of individual studies have been conflicting and hence we conducted a systematic review and meta-analysis to combine existing data on the efficacy and tolerability of MRA in LRH.

## Methods

### Eligibility criteria

RCTs of adults with LRH comparing MRA versus placebo or other antihypertensives were included. Outcomes of interest were a) change in BP, b) time to target BP, c) defined daily dose of antihypertensive required to achieve target BP, d) end-organ dysfunction and e) adverse effects. Participants with a known secondary cause of hypertension including PA or monogenic causes of LRH, records not in English language and conference abstracts were excluded. The protocol for this review is registered in the international registry, PROSPERO (CRD42022318763).

### Search strategy and selection process

A systematic search based on the selection criteria and combining Medical Subject Headings and text words was developed using the OVID platform (Supplementary Table 1). Medline, EMBASE and Cochrane databases were searched (SS) to identify records from inception to 19/12/22. Two independent reviewers (SS, JZ) reviewed the records retrieved. Full texts were sought if initial screening suggested that the study met the selection criteria. Any disagreement was resolved by a third reviewer (JY).

### Data extraction and risk of bias assessment

Using a template designed in Covidence, author information, study design, participant characteristics, LRH definition, intervention, results, compliance, and dropout rates were extracted [12]. For records published after 2000, authors were contacted to request trial protocols and outcomes that were not reported. Any discordant extracted data was discussed, and a consensus was reached (SS, JZ, JY).

The methodological quality was assessed by two independent reviewers (SS, JZ) using the Cochrane risk of bias tool 2 for RCT [13]. Discordant assessments were settled by consensus (SS, JZ, JY).

### Effect measures and data synthesis

The change in BP was reported as the mean difference in SBP, DBP, or mean arterial pressure (MAP) from baseline to the last time point at which BP was measured. Studies were grouped based on comparator drug classes. Data were presented as mean and standard deviation (SD). Missing SD values were obtained using methods described in the Cochrane Handbook [14]. Where only baseline and post-treatment SD were available, SD for the change in BP were imputed using correlation coefficient values derived from a study with a similar design that reported individual participant data. Where no measure of variance was reported, the mean of the SD from studies with the same drug class comparator was used to estimate the SD for that study. Supine and erect BP SD were pooled [15]. A meta-analysis was performed if data for change in SBP or DBP for ≥3 studies were available. If studies compared ≥1 dose of antihypertensive, the BP result from the highest dose was included in the meta-analysis. Aggregated mean treatment group difference (mean change in blood pressure with MRA minus mean change in blood pressure with comparator) and 95%-confidence intervals (95% CI) were calculated using an inverse-variance method using Review Manager software version 5.4 [16]. A random effect model was chosen based on the assumption that there was methodological diversity that would impact the effect of the intervention among the studies. Statistical significance was set at p < 0.05. Statistical heterogeneity, measuring between-study variation, was quantified using the I^2^ test and interpreted as suggested in the Cochrane Handbook [17]. Publication bias was assessed using a funnel plot if there were ≥5 studies. Subgroup analyses were not conducted due to the small number of studies in each meta-analysis. Sensitivity analysis using the leave-one-out method, removal of high-risk of bias studies and using different correlation coefficients for imputed SD was performed to test the underlying assumptions. Adverse effects were reported as percentages.

### Certainty assessment

The Grading of Recommendations, Assessment, Development, and Evaluation (GRADE) approach was used to evaluate the certainty of evidence (SS) [18].

Results are reported according to the Preferred Reporting Items for Systematic Reviews and Meta-Analyses (PRISMA) guidelines [19].

## Results

**Fig. 1:**
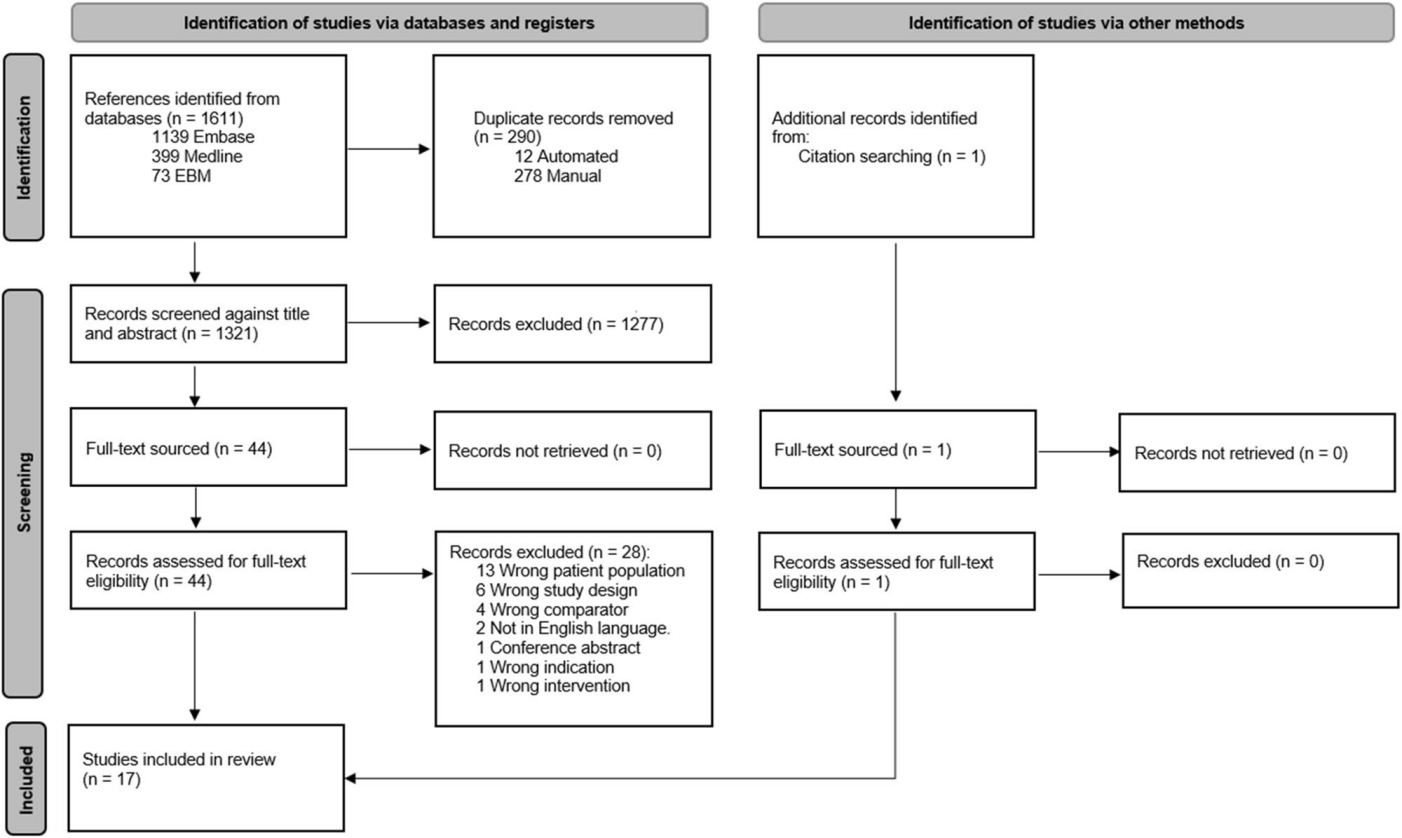
PRISMA flow chart. Flow chart of selection of randomised controlled trials included in this systematic review.

The database search identified 1611 records and 1 record was identified by searching the references of the included articles (Fig. 1). Seventeen studies with 1043 participants published between 1972 and 2007 were deemed eligible for inclusion in this systematic review and summarised in Table 1. The rest were excluded as they did not meet our PICO criteria; incorrect population (not low-renin hypertension), intervention (not a randomised controlled trial assessing effect of MRA), comparison (not placebo or active drug) or outcome (change in BP, end-organ dysfunction or adverse effects were not reported). Ten studies were cross-over studies and seven were parallel. All studies except two were blinded. In nine studies, LRH was a subgroup of the trial population. The median duration of intervention was eight weeks (range of four to twenty-four weeks). Eleven studies reported support from or an affiliation with a pharmaceutical company [20–30]. MRA therapy was compared to diuretics in nine studies, angiotensin-converting enzyme inhibitors and angiotensin receptor blockers (ACEi/ARB) in four studies, epithelial sodium channel inhibitors (ENaCi) monotherapy in three studies, placebo in two studies and a beta-blocker and an alpha-2 agonist in one study [20–36].

**Table 1:** Baseline characteristics of studies included in the systematic review.

Study	Site	Study design	Number of participant	LRH criteria	Renin			PA excluded	Mean age, years (SD)	Male %	Ethnicity % White	Intervention and comparator		Rx Time (wks)
					Mean renin (SD)	Assay	Measured off BP agents							
Adlin 1972	USA	Parallel; Blinding unclear	Subgroup; 28 (Sp:18, Hct:10)	Low-normal renin groups pooled together Stimulated PRA < 400 ng/100 ml + 4 hr upright + 3-day LSD	223 ng /100 ml	PRA -immunoassay for angiotensin II	Yes	NR	Sp:46 Hct:42	Sp:28 Hct:30	Sp:28 Hct:0	Sp 50 mg QID	Hct 25 mg QID	12
Carey 1972	USA	Crossover; Double-blinded	Subgroup; 24	Stimulated PRA < 500 ng/100 ml/3 hr x3 doses furosemide 40 mg 6hrly+ 3 hr upright	184.8 (145.1) ng/100 ml/3 h	PRA -immunoassay for angiotensin I	Yes	Yes. 24-hr urine aldo excretion	47.0 (9.5)	38	29	Sp 400 mg	Placebo	6
Vaughan 1973	USA	Parallel; open-labelled	Subgroup; 37 (Sp:21, Ch:16)	Below nomogram values of PRA versus 24-hr urine sodium excretion of healthy subjects whilst on LSD	0.8 (0.4) ng/ml/hr	PRA -immunoassay for angiotensin I	Yes	NR	54.4 (10.2)	Sp:62 Ch:38	Sp:67 Ch:69	Sp 50–400 mg	Ch 50–100 mg	6
Douglas 1974	USA	Crossover; Double blinded	17	Stimulated PRA < 1.67 ng/ml/hr x3 furosemide 40 mg 6hrly, 3 hr upright	NR range <0.2–1.4 ng/ml/hr	PRA -immunoassay for angiotensin I	Yes	Yes. 24-hr urine aldo excretion	49.9 (8.6)	47	35	Sp 100 mg QID	Hct-Tr 25/50 mg QID	6
Spark 1974	USA	Crossover; Double blinded	10	Stimulated PRA < 3.5 ng /ml/hr after 4 hr upright posture and <7 ng/ml/hr after 3 hr post oral furosemide 80 mg whilst on a 120-mEq Na and 40-mEq K+ diet	NR	PRA -immunoassay for angiotensin I	Yes	Yes, method NR	37	NR	NR	Sp 100 mg QID	Hct 50 mg QID	5
Hunyor 1975	USA	Crossover; Double blinded	Subgroup;12	Stimulated PRA < 50 ng%/hour on LSD 7d and 2 hr ambulation	25.3 (10.2) ng%hr	PRA -immunoassay for angiotensin I	Yes	NR	NR for the subgroup. WG 43(range 20–59)	75	50	Sp 200–400 mg	Ch 100 mg	4
Thomas 1976	UK	Crossover, titration to effect; Double blinded	Subgroup;15	Unstimulated lowest tertile of supine PRA on an unrestricted salt diet	NR Range 0.4–1.1 ng/ml/hr	PRA -immunoassay for angiotensin I	Yes	NR	NR	NR	100	Sp 200–400 mg	Ox 160–640 mg Me 750–3000 mg	8
Brooks 1977	USA	Crossover, titration to effect; Double blinded	Subgroup; 9	Stimulated PRA < 0.8 ng/ml/hr after an overnight fast and 4 hr after oral furosemide 80 mg and an upright posture	Sp: PRA 0.2 (0.1) ng/ml/hr Hct: PRA 0.4 (0.2) ng/ml/hr	PRA – method NR	NR	NR	45	21	11	Sp 25–100 mg BD	Hct 25–100 mg BD or Sp/Hct 25/25–100/100 mg BD and 50/50–200/200 mg daily	6
Ferguson 1977	USA	Crossover; Double blinded	Subgroup;11	PRA ≤ 1.0 ng/ml/hr on LSD diet and 2 hr upright posture	0.4(0.1) ng/ml/hr	PRA -immunoassay for angiotensin I	Yes	NR	NR	NR	NR	Sp 100 mg QID	Hct 25 mg QID	6
DeCarvalho 1980	USA	Crossover, titration to effect; Double blinded	13	Low PRA (for sodium intake) and an expanded plasma volume, cut-off value not defined	0.8(0.9) ng/ml/hr	PRA	Yes	NR	49	23	NR	Sp 25 mg QID titrated weekly	Tr 25 mg QID titrated weekly	8
Kreeft 1983	Canada	Crossover; Double blinded	19	Stimulated PRA < 3 ng/ml/hr post oral furosemide 40 mg, 4 hr upright	1.2(0.8) ng/ml/hr	PRA -immunoassay for angiotensin I	Yes	NR	NR (Range 42–66 years)	53	100	Sp 400 mg	Ch 100 mg	8
Flack 2003	South Africa and USA	Parallel, titration to effect; Double blinded	Subgroup; NR ~ 183	Lowest tertile of active renin (<8.2mU/L)	NR	Active renin measured by radioimmunoassay	Yes	NR	NR	NR	NR	Ep 50–200 mg	Lo 50–100 mg	16
Saruta 2004	Japan	Parallel; Double-blinded	193 (Placebo:50, Ep50:49 Ep100:46 Ep200: 48)	Not a study inclusion criterion but baseline active plasma renin levels were consistently low	5.7–10.1 mU/L (for each group)	Active renin concentration	Yes	NR	Ep50: 54.2 (11.3) Ep100:52.8 (10.0) Ep200:52.6 (10.8) Placebo: 54.3 (10.6)	Ep50:63 Ep100:70 Ep200:73 Placebo:68	0	Ep 50–200 mg	Placebo	8
Williams 2004	USA, Canada Germany, Spain	Parallel; Double-blinded	Subgroup;149 (Ep:67, En:82)	Lowest tertile of unstimulated active renin (<7.2 pg/ml)	NR	Active renin – immunoassay	Yes	NR	NR	NR	NR	Ep 50–200 mg	En 10–40 mg	24
Saha 2005	USA	Parallel; Double-blinded	98 (Sp:23, Am:26, SpAm:22, Placebo: 27)	Unstimulated PRA < 0.56 ng/L/sec	Sp:0.3(0.5) Am:0.2(0.6) Sp/Am:0.1(0.2) Placebo:0.2(0.3) Ng/L/sec	PRA-radioimmunoassay for angiotensin I	No (n = 20 on βblockers and n = 89 on diuretics)	No	Sp: 48.5(8.9) Am: 44.5(9.4) SpAm: 46.3(9.2) Placebo: 46.4(9.4	Sp:48 Am:54 SpAm:59 Placebo:52	0	Sp 25 mg	Am 10 mg, combination, placebo	9
Weinberger 2005	France, Spain, UK and USA	Parallel; titration-to- effect; Double blinded	168 (Ep:86, Lo:82)	Unstimulated PRA ≤ 1.0 ng/mL/hr or active renin value ≤ 25 pg/mL ( ≤ 42.5mU/L)	Ep:11.6 mU/L Lo:12.7mU/L	Active renin concentration	Yes	Yes, method NR	Ep: 54.2 (9.7) Lo:53.9 (10.2)	Ep:47 Lo:56	NR (% Nonblack Ep: 67 Lo:62)	Ep 100–200 mg	Lo 50–100 mg	8
Hood 2007	UK	Crossover; Double blinded	57	Unstimulated renin≤12 mU/L ( ≈ 0.65 ng/mL/hr), normal potassium, ARR > 750, previous response to Sp( > 20 mmHg SBP reduction)	NR Median 6.3 mU/L (range 3.4–13.0)	Renin mass	Yes	No	59.5(11.9)	54	98	Sp 50–100 mg	Be 2.5–5 mg, Am 20–40 mg or Lo 100 mg and non-diuretic and placebo control	5

*Am* amiloride, *α* alpha, *aldo* aldosterone, *Be* Bendroflumethiazide, *β* beta, *Ch* chlorthalidone, *DBP* diastolic blood pressure, *En* enalapril, *Ep* eplerenone, *Hct* hydrochlorothiazide, *Hr* hour, *Lo* losartan, *LSD* low salt diet, *Me* methyldopa, *MRA* mineralocorticoid receptor antagonist, *NR* not reported, *Ox* oxprenolol, *PRA* plasma renin activity, *Rx* treatment, *SBP* systolic blood pressure, *SD* standard deviation, *Sp* spironolactone, *Tr* triamterene. *UK* United Kingdom, *USA* United States of America, *WG* whole group, *wks* weeks.

Seven studies were assessed as having a high risk of bias and the other ten studies were judged to have some concerns (Supplementary Table 2). Most trials did not have a trial protocol registered prospectively and allocation concealment was not reported [20–29, 31–36]. A potential carry-over effect of previous treatment was identified in three cross-over studies [30, 33, 34]. The attrition rate was incompletely reported, especially in the studies in which LRH was a subgroup. Two out of the eight studies that reported dropout rates had high attrition of participants (>20%) [29, 36].

### Blood pressure

BP results are summarised in Supplementary Table 3. Fourteen studies reported changes in mean SBP and DBP. The remaining studies only reported a change in MAP, defined as DBP + pulse pressure/3.

### MRA versus diuretics

Six studies reported changes in mean SBP and DBP: four cross-over and two parallel studies [20–22, 25, 30, 32]. Treatment duration ranged from four to twelve weeks. Spironolactone (50–400 mg/day) was compared to chlorthalidone (50–100 mg/day), hydrochlorothiazide (100–200 mg/day) or bendroflumethiazide (5 mg/day) (Table 1). One study compared spironolactone to a combination medication hydrochlorothiazide/triamterene [21]. Triamterene, an ENaCi, is often used as an adjunct therapy due to its potassium-sparing properties but not as monotherapy due to its weak BP-lowering effect [37]. Nevertheless, given that MRA also reduces ENaC activity, we have excluded this study from the meta-analysis to ensure clear comparisons of the different classes of medication.

**Fig. 2:**
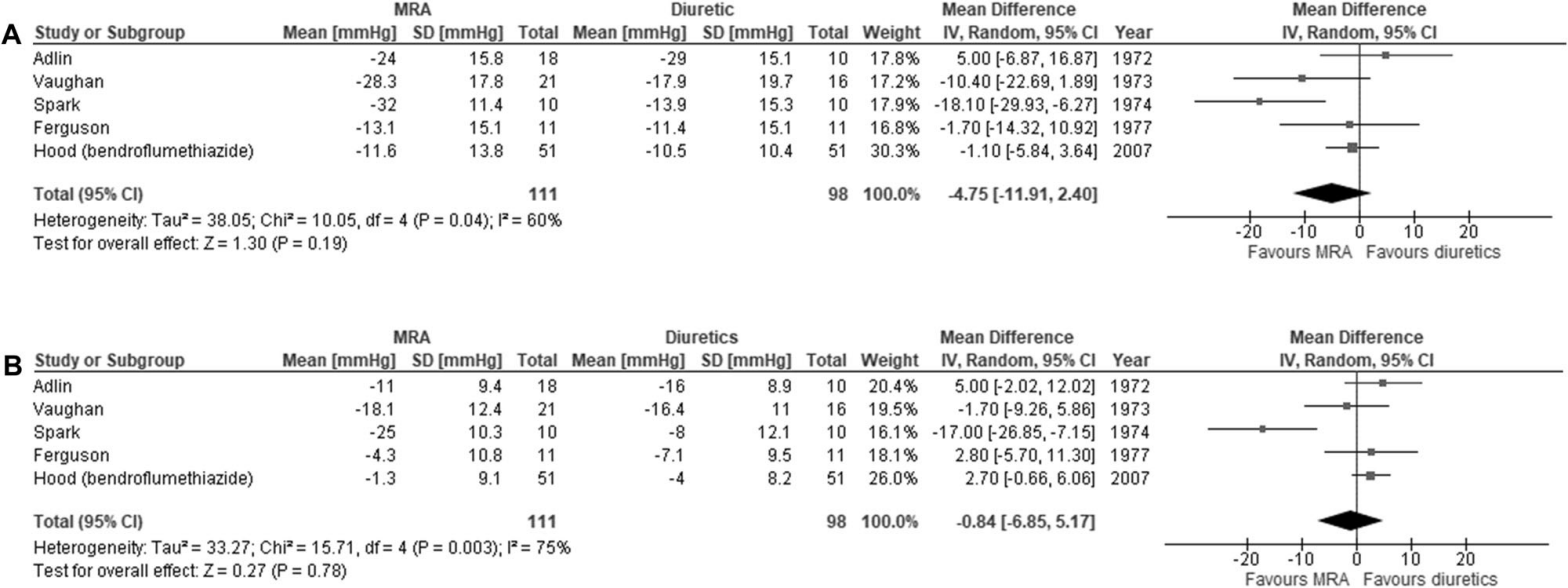
Meta-analysis of blood pressure lowering effect with mineralocorticoid receptor antagonists (MRA) versus diuretics. **A** systolic blood pressure; **B** diastolic blood pressure.

The mean difference in SBP change between MRA (n = 111) and diuretics (n = 98) was −4.8 mmHg (95% CI −11.9, 2.4) with moderate heterogeneity between studies (I^2^ = 60%, p = 0.04) (Fig. 2). Sensitivity analysis using the leave-one-out method increased the aggregated mean difference in SBP between MRA and diuretics to −6.9 mmHg (95% CI −15.0, 1.2) when the Adlin *et al* study was removed (Supplementary Table 4) [20]. Whereas removal of the Spark *et al* study resulted in a reduction in heterogeneity, with I^2^ decreasing from 60% to 6%, and a smaller aggregated mean difference of −1.5 mmHg (95% CI −5.8, 2.8) [22]. Further sensitivity analysis using different correlation coefficients (±0.2) used to calculate the imputed SD for this study did not reveal a difference in effect measure (Supplementary Table 5). Removal of high-risk of bias studies (N = 3) increased the mean difference to −10.1 mmHg (95% CI −26.1, 6.0). Visual inspection revealed a symmetrical funnel plot (Supplementary Fig. 1).

The mean difference in DBP change was −0.8 mmHg (95% CI −6.9, 5.2) with substantial heterogeneity detected between studies (I^2^ = 75%, p < 0.01) (Fig. 2). Sensitivity analysis for change in DBP with the removal of high-risk of bias studies (N = 3) increased the aggregated mean difference to −6.9 mmHg (95% CI −26.3, 12.5). The certainty of the evidence was rated to be very low for the difference in SBP and DBP outcomes (Table 2).

**Table 2:** Summary of findings for blood pressure lowering effect of mineralocorticoid receptor antagonists versus comparator meta-analysis.

Outcomes	Number of participants, n (Number of studies, N)	Aggregated mean difference in blood pressure^a^ mmHg (95% CI)	Quality of evidence (GRADE)	Comments
MRA versus diuretics on SBP	MRA: n = 111 Diuretic: n = 98 (N = 5) [20, 22, 25, 30, 32]	−4.8 (−11.9, 2.4)	⨁◯◯◯ **Very low**	3/5 studies high risk of bias, moderate inconsistency: I^2^ = 60% (p = 0.04), imprecision: CI includes the possibility of no effect and benefit with MRA
MRA versus diuretics on DBP	MRA: n = 111 Diuretic: n = 98 (N = 5) [20, 22, 25, 30, 32]	−0.8 (−6.9, 5.2)	⨁◯◯◯ **Very low**	3/5 studies high risk of bias, substantial inconsistency: I^2^ = 75% (p < 0.01), imprecision: CI includes the possibility of no effect and benefit with MRA
MRA versus ACEi/ARB inhibitor on SBP	MRA: n = 264 ACEi/ARB: n = 277(N = 4) [26, 28–30]	−6.8 (−9.6, −4.1)	⨁⨁⨁◯ **Moderate**	2/4 studies high risk of bias
MRA versus ACEi/ARB inhibitor on DBP	MRA: n = 264 ACEi/ARB: n = 277 (N = 4) [26, 28–30]	−2.5 (−5.9, 1.0)	⨁◯◯◯ **Very low**	2/4 studies high risk of bias, considerable inconsistency: I^2^ = 83% (p < 0.01), imprecision: CI includes the possibility of no effect and benefit with MRA
MRA versus ENaCi on SBP	MRA: n = 87 ENaCi: n = 90 (N = 3) [30, 34, 36]	−0.9 (−9.0, 7.1)	⨁◯◯◯ **Very low**	2/3 studies high risk of bias, considerable inconsistency: I^2^ = 77% (p = 0.01), imprecision: CI includes the possibility of no effect and benefit with MRA and ENaCi
MRA versus ENaCi on DBP	MRA: n = 87 ENaCi: n = 90 (N = 3) [30, 34, 36]	1.45 (−0.7, 3.6)	⨁⨁◯◯ **Low**	2/3 studies high risk of bias, imprecision: CI includes the possibility of no effect and benefit with ENaCi

*ACEi/ARB* angiotensin-converting enzyme inhibitors/angiotensin receptor blockers, *CI* confidence interval, *DBP* diastolic blood pressure, *ENaCi* epithelial sodium channel inhibitors, *MRA* mineralocorticoid receptor antagonists, *SBP* systolic blood pressure.

^a^Aggregated mean treatment group difference was calculated by mean change in blood pressure with MRA minus mean change in blood pressure with the comparator.

### MRA versus ACEi/ARB

**Fig. 3:**
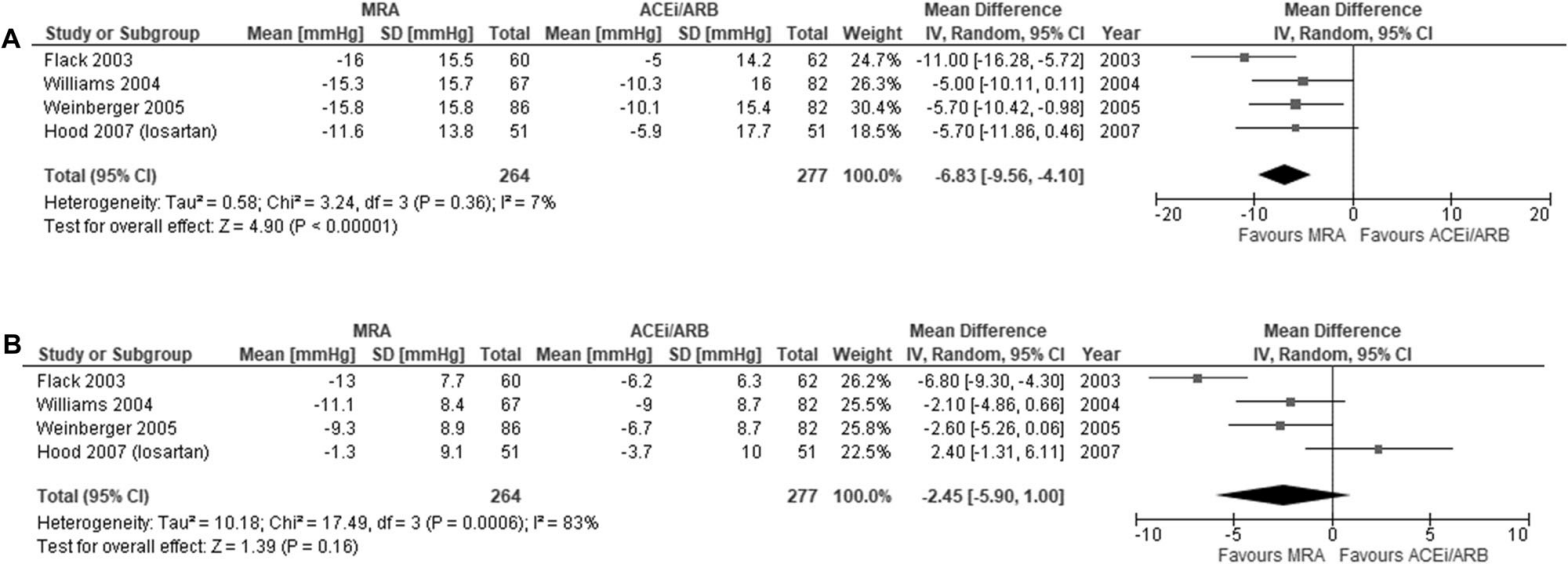
Meta-analysis of blood pressure lowering effect with mineralocorticoid receptor antagonists (MRA) versus angiotensin-converting enzyme inhibitors/angiotensin receptor blockers (ACEi/ARB). **A** systolic blood pressure; **B** diastolic blood pressure.

Four studies reported changes in mean SBP and DBP: one cross-over and three parallel studies [26, 28–30]. Treatment duration ranged from five to sixteen weeks. Spironolactone (50–100 mg/day) or eplerenone (50–200 mg/day) were compared to losartan (50–100 mg/day) or enalapril (10–40 mg/day). The mean difference in change in SBP between MRA (n = 264) and ACEi/ARB (n = 277) was −6.8 mmHg (95% CI −9.6, −4.1) with low heterogeneity between studies (I^2^ = 7%, p = 0.36) (Fig. 3).

The mean difference in DBP change was −2.5 mmHg (95% CI −5.9, 1.0) with considerable heterogeneity between studies (I^2^ = 83%, p < 0.001) (Fig. 3). Sensitivity analysis with the removal of high risk of bias studies (N = 2) reduced heterogeneity (I^2^ = 0, p = 0.80) with an aggregated mean difference in DBP of −2.4 mmHg (95% CI −4.3, −0.4) (Supplementary Table 6). The certainty of the evidence for the difference in SBP and DBP was rated moderate and very low respectively (Table 2).

### MRA versus ENaCi

**Fig. 4:**
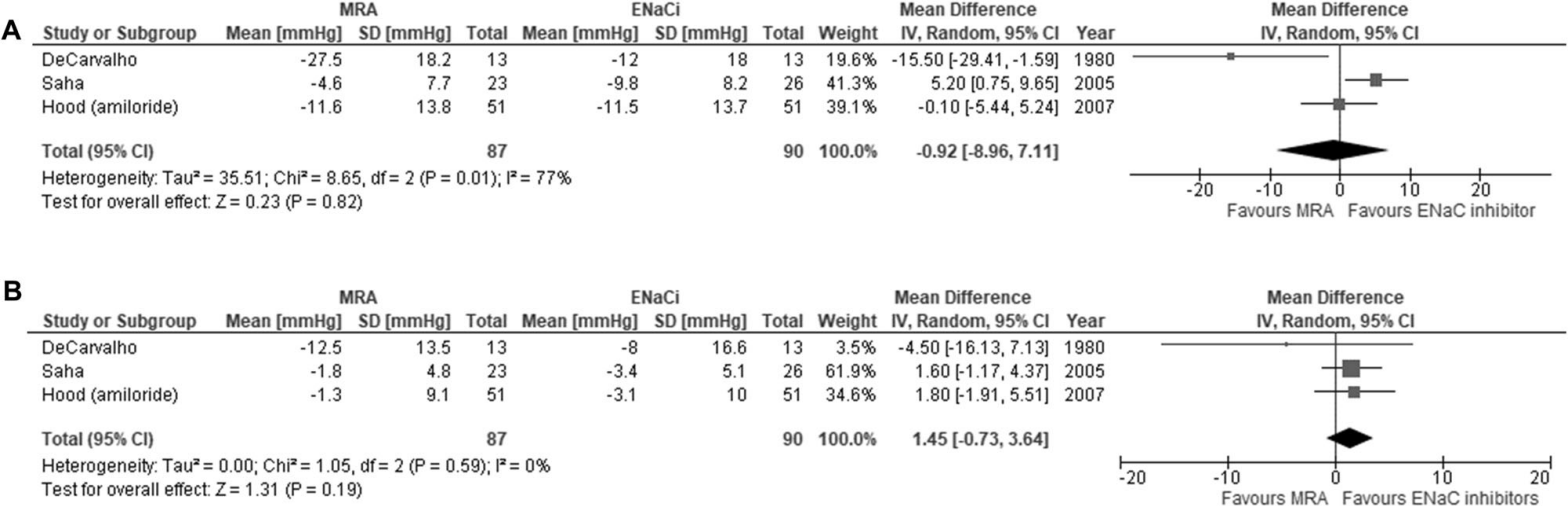
Meta-analysis of blood pressure lowering effect with mineralocorticoid receptor antagonists (MRA) versus epithelial sodium channel inhibitors (ENaCi). **A** systolic blood pressure; **B** diastolic blood pressure.

There were three studies comparing spironolactone (25 mg/day to mean 224 mg/day) with amiloride (10–40 mg/day) or triamterene monotherapy (mean dose 268 mg/day) [30, 34, 36]. The mean difference in SBP change between MRA (n = 87) and EnaCi (n = 90) was −0.9 mmHg (95% CI −9.0, 7.1) with considerable heterogeneity (I^2^=77%, p = 0.01) (Fig. 4). Sensitivity analysis with the removal of the high risk of bias studies (N = 2) increased the mean difference in SBP (N = 1) to 5.2 mmHg (95% CI 0.8, 9.7) (Supplementary Table 7).

The mean difference in DBP change was 1.5 mmHg (95% CI −0.7,3.6) with low heterogeneity between studies (I^2^ = 0%, p = 0.59) (Fig. 4). A sensitivity analysis with the removal of each study, high risk of bias studies (N = 2) and using different correlation coefficients (±0.2) to calculate imputed SD did not reveal a difference in aggregated mean DBP (Supplementary Tables 7–8). The certainty of the evidence for the difference in SBP and DBP outcomes was rated very low and low respectively (Table 2).

### MRA versus placebo

Two studies compared MRA to placebo treatment; MRA was effective in reducing SBP and DBP compared to placebo. One study was a cross-over study comparing spironolactone 400 mg to placebo (n = 24) for six weeks [31]. The differences in mean change in SBP and DBP between spironolactone 400 mg and placebo were −33.4 mmHg (95% CI −40.4, −26.4) and −15.8 mmHg (95% CI −19.8, −11.8), respectively. The second study was a parallel study comparing eplerenone 50 mg (n = 49), 100 mg (n = 46) and 200 mg (n = 48) to placebo (n = 50) for eight weeks (Table 1) [27]. The differences in mean change in SBP and DBP between eplerenone 200 mg compared to placebo were −8.5 mmHg (95% CI −10.8, −6.2) and −4.5 mmHg (95% CI −6.7, −2.3), respectively.

### MRA versus beta-blocker and alpha-2 agonist

One cross-over study compared spironolactone 200–400 mg to oxprenolol 160–640 mg and methyldopa 750–3000 mg for eight weeks [23]. The difference in SBP change between treatment groups was −13.5 mmHg and −13.0 mmHg respectively (Supplementary Table 3). The difference in DBP change was −3.1 mmHg and −0.8 mmHg. SD or CI was not reported.

### Adverse effects

Adverse effects were reported in seven studies and summarised in Table 3.

**Table 3:** Adverse effects reported in the studies.

Drug class	Study	Intervention	Adverse effects (MRA)	Adverse effects (comparator)
MRA versus diuretics	Douglas 1974	Sp 400 mg compared to Hct 100+Tr 200 mg	Breast tenderness 11.7% Amenorrhoea 5.9% Impotence 11.7% Muscle cramps 17.6% Lassitude 23.5% Hypokalaemia 0% Angina and hypotension 0%	Breast tenderness 0% Amenorrhoea 0% Impotence 0% Muscle cramps 23.5% Lassitude 23.5% Hypokalaemia 5.9% Angina and hypotension 5.9%
	Spark 1974	Sp 400 mg compared to Hct 200 mg	None	Hypokalaemia and muscle cramps (% NR)
	Kreeft 1983	Sp 400 mg compared to Ch 100 mg	Orthostatic dizziness 16%	Orthostatic dizziness 0%
MRA versus ACEi/ARB	Weinberger 2005	E100–200mg compared to Lo 50–100 mg	Gynaecomastia 2.3% Irregular menses 0% Impotence 1.2% Hyperkalaemia: 1.2%	Gynaecomastia 0% Irregular menses 2.4% Impotence 1.2% Hyperkalaemia: 0%
MRA versus ENaCi	DeCarvalho 1980	Sp 100-mg compared to Tr 100-mg	Orthostatic dizziness and hypotension 8%	Orthostatic dizziness and hypotension 0%
MRA versus placebo	Carey 1972	Sp 400 mg compared to placebo	Breast tenderness 0% Impotence 12.5% Muscle cramps 8.3% General weakness 4.2%	Breast tenderness 0% Impotence 0% Muscle cramps 0% General weakness 0%
	Saruta 2004	Ep 50,100,200 mg compared to placebo	Breakdown NR. No difference between groups.	

*ACEi/ARB* angiotensin-converting enzyme inhibitors/angiotensin receptor blockers; *Ch* chlorthalidone, *ENaCi* epithelial sodium channel inhibitors, *Ep* eplerenone, *Hct* hydrochlorothiazide, *Lo* losartan, *MRA* mineralocorticoid receptor antagonist, *NR* not reported, *Sp* spironolactone; *Tr* triamterene.

### Time to target BP, defined daily dose of medication required to achieve target BP and end-organ dysfunction

These outcomes were not reported in any of the studies.

## Discussion

This systematic review and meta-analysis suggest that MRA are more effective at lowering BP in LRH compared to placebo and ACEi/ARB, particularly for SBP. Two studies with follow-ups of more than three months suggest that this difference in the BP-lowering effect may be maintained [26, 28]. There was a trend towards favouring MRA use over ACEi/ARB for lowering DBP as well. This was significant when studies assessed to be high risk of bias were removed. This supports the notion that further suppression of the renin-angiotensin system is less effective in a low-renin state compared to blocking MR activation. This is an important finding as ACEi/ARB are commonly prescribed first-line anti-hypertensives [1, 4]. It is possible that this differential BP response to MRA would have been strengthened if more studies with longer follow-up were available due to the aldosterone escape phenomenon described with the chronic use of ACEi [38].

In the MRA versus diuretic meta-analysis, there was a trend towards favouring MRA lowering SBP compared to thiazide and thiazide-like diuretics. This supports the hypothesis that in addition to an excess salt/volume state, there is inappropriate MR activation in many people with LRH [10, 39]. This difference in efficacy was increased when data from the Adlin *et al* study, which had a more relaxed LRH definition including low-normal renin, was removed [20]. Thiazide and thiazide-like diuretics may be a preferable second or third-line antihypertensive for LRH compared to beta-blockers or ACEi due to their natriuretic effect. Turner *et al* reported that in 363 participants with essential hypertension, a lower plasma renin activity (PRA) predicted a better blood pressure-lowering response to hydrochlorothiazide (12.5–25 mg/day) compared to atenolol (50–100 mg/day), both as monotherapy and as an add-on [40]. In a retrospective analysis, among 313 participants with PRA in the lowest tertile (<0.74 ng/ml/h), it was hypothesised excess sodium and expanded volume contributed to hypertension, natriuretic anti-hypertensives, diuretics and calcium channel blockers, were more effective in lowering SBP (−16 versus −6 mmHg, p < 0.001) and DBP (−8 versus −5mmHg, p = 0.008) compared to renin-angiotensin targeting anti-hypertensives, beta-blockers and ACEi [41].

The meta-analysis of MRA versus ENaCi revealed no differences in the BP-lowering efficacy in LRH. This may be due to amiloride and triamterene having a direct inhibitory effect on ENaC, which is a downstream target of the MR [42]. Amiloride is the preferred ENaCi as triamterene is a weak anti-hypertensive associated with rare but serious side effects including nephrolithiasis, interstitial nephritis and drug hypersensitivity [37, 43, 44]. However, in conditions with inappropriately high aldosterone concentration, ENaCi may not confer the same cardiovascular protection as MRA given that MR expression regulates cardiac and vascular tissue remodelling via activation of other cellular targets [45, 46].

In addition, it is possible that people with Liddle’s syndrome, who have increased ENaC activity due to a gain of function genetic mutation in the epithelial sodium channel subunits, were not excluded in the trial populations [47]. Though monogenetic Liddle syndrome is rare, a more common and less severe phenotype has been described by Spence *et al*, characterised by low renin, low aldosterone and responsiveness to amiloride treatment [48]. A prospective study in Africa found that personalising treatment based on both renin and aldosterone concentrations improved BP control compared to usual care (50% versus 11% achieved BP < 140/90 mmHg in the respective groups) [49]. In this approach, participants with low renin-high aldosterone concentrations were treated with MRA, whereas those with low renin-low aldosterone were treated with amiloride and high renin-high aldosterone were treated with ARB.

There are three main limitations of this systematic review and meta-analysis. The population was heterogeneous due to different definitions of LRH. Some authors defined low renin as a value below a prespecified level after stimulation with low salt intake, erect posture and/or diuretics [20–22, 24, 25, 31–33, 35]. In contrast, others defined low renin as the lowest tertile of measured renin in a trial population with essential hypertension [23, 26, 28]. In one study done in a Japanese population with hypertension, although low renin was not a study inclusion criterion, authors report that the majority of participants had low renin (mean active plasma renin ranged from 5.7 mU/L to 10.1 mU/L in the different treatment groups) [27]. The method of measuring renin varied as well; some studies used PRA whereas other studies used direct renin concentration (DRC). Conversion factors of PRA (ng/mL/h) to DRC (mU/L) of 8.2–12 have been suggested but do not correlate well in the range of interest (PRA < 1 ng/ml/h) or under conditions such as in the presence of high estrogen (lower DRC), congestive heart failure (lower PRA) and concomitant direct renin inhibitor treatment (lower PRA and higher DRC) [50, 51]. One study reported measuring renin whilst some participants were on beta-blockers, which can falsely lower renin levels [36]. As such, some participants may have been incorrectly classified as having LRH and therefore confounded any potential differences in the response to treatment. Sensitivity analysis by removing this study increased the mean aggregated difference in SBP in the MRA versus ENaCi meta-analysis but did not reach statistical significance (−6.4 mmHg, 95% CI −21.3, 8.4). Furthermore, PA was not rigorously excluded. Some participants may have undiagnosed PA, which would respond favourably to MRA therapy. PA was excluded using tests with low sensitivity; the presence of hypokalaemia (up to 95% are normokalaemia) and elevated 24-hour urinary aldosterone excretion (accuracy of results depends on whether the sample is collected correctly) [52]. One study included participants who had a previous BP-lowering response to spironolactone (>20 mmHg SBP reduction) [30].

A further limitation of this meta-analysis is that multiple different antihypertensives and doses were compared; the BP-lowering effect of MRA and comparator dose may not be equipotent. Furthermore, current antihypertensive prescribing practices have changed; some of the comparators are no longer routinely used or are utilised in much lower doses [1, 4]. Importantly, no studies compared MRA to calcium channel blockers, a common class of antihypertensives prescribed for essential hypertension in current practice. In addition, the treatment effect measured may have been confounded by the short or absent washout period in some cross-over studies [30, 33, 34].

Translation to clinical practice is also limited by incomplete assessment of the tolerability of treatment and their impact on end-organ function. Only six out of seventeen studies reported specific adverse effects and the longest duration of follow-up was only nine weeks. Potential dose-dependent adverse effects that may limit the use of MRA include hyperkalaemia and the progestogenic and anti-androgenic effects of spironolactone (breast tenderness, gynecomastia, oligomenorrhea, and sexual dysfunction) [53]. More selective MRA, such as eplerenone, with low affinity for progesterone and androgen receptors are better tolerated and useful for patients with PA and LRH [54]. In PA, MRA has a cardioprotective effect that is independent of its BP-lowering effect [7]. Studies in this systematic review did not report on measures of end-organ function and therefore, it is not known whether the use of MRA reduces cardiovascular risk in patients with LRH. This would be of interest given that data from a large observational study suggested that patients with hypertension and low renin have an increased cardiovascular risk profile compared to those with normal renin [55].

## Conclusion

A pathophysiology-based approach to the management of hypertension is promising and may be key to addressing the burden of hypertension. MRA therapy is effective in lowering blood pressure in LRH and may be better than ACEi/ARB. However, further RCTs with a rigorous methodology addressing the limitations highlighted in this review are needed to accurately assess the benefits and risks. Studies with a longer follow-up, data on tolerability and markers of end-organ dysfunction comparing lower dose spironolactone, selective MRA or ENACi to contemporary antihypertensives are needed to support the recognition of LRH as a subtype of hypertension with targeted treatment options.

## Summary

### What is known about the topic

- Low-renin hypertension is common and affects one in three patients with hypertension.
- The underlying disease process for a large proportion of patients with low-renin hypertension is largely undefined and the optimal treatment is not known.

### What this study adds

- Our systematic review and meta-analysis found that in low-renin hypertension, treatment with mineralocorticoid receptor antagonists lowered systolic blood pressure to a greater extent compared to commonly used first-line antihypertensive agents such as angiotensin-converting enzyme inhibitors and angiotensin receptor blockers, and to a similar extent when compared to epithelial sodium channel inhibitors.
- As such, targeted treatment with mineralocorticoid receptor antagonists should be considered in people with low-renin hypertension and epithelial sodium channel inhibitors may be considered as an alternative treatment.
- Results of our meta-analysis suggest that the underlying pathophysiology in a large proportion of people with low-renin hypertension is one of excess salt, volume expansion and/or mineralocorticoid receptor activation.

### Supplementary information

Supplementary material

## Data Availability

Some or all datasets generated during and/or analysed during the current study are not publicly available but are available from the corresponding author on request.
