# Supplementary material for: Efficacy and safety of mineralocorticoid receptor antagonists for the treatment of low-renin hypertension: a systematic review and meta-analysis
Source: J Hum Hypertens. 2024 Jan 11;38(5):383–92. doi: 10.1038/s41371-023-00891-1 (PMC11076210; doi:10.1038/s41371-023-00891-1)
Supplement: Supplementary file 1 — Supplementary material [file 41371_2023_891_MOESM1_ESM.docx]

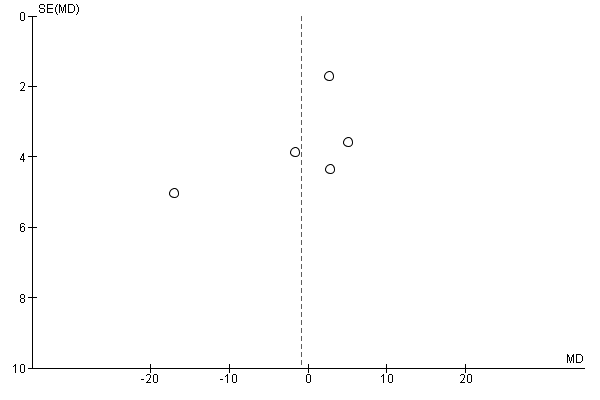


**SE(MD)**

0

2

4

6

8

10

**SE(MD)**

0

2

4

6

8

10

**B**

**A**


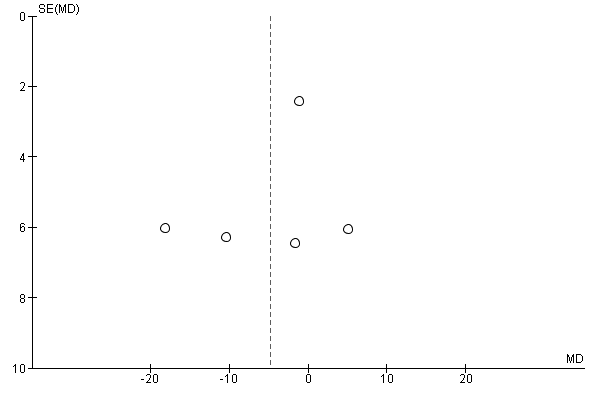


-20 -10 0 10 20 **MD**

-20 -10 0 10 20 **MD**

Supplementary Figure 1: Mineralocorticoid receptor antagonists versus diuretics effect on systolic blood pressure (A) and diastolic blood pressure (B) funnel plots.

The x-axis represents the mean treatment group difference, calculated by the mean change in blood pressure with mineralocorticoid receptor antagonists minus the mean change in blood pressure with diuretics (MD); the y-axis represents the standard error of the mean treatment group difference (SE(MD)).

Supplementary Table 1: Search strategy

| 1. | blood pressure/ |
| --- | --- |
| 2. | hypertension/ |
| 3. | 1 or 2 |
| 4. | renin/ or plasma renin activity/ or renin angiotensin disorder/ or renin angiotensin aldosterone system/ |
| 5. | 3 and 4 |
| 6. | ((hyperten* or elevated blood pressure or high blood pressure) and (suppressed renin or low renin or (low adj3 renin))).mp. |
| 7. | 5 or 6 |
| 8. | mineralocorticoid receptor*.mp. or mineralocorticoid receptor/ |
| 9. | mineralocorticoid antagonist*.mp. or mineralocorticoid antagonist/ |
| 10. | adrenal blocker*.mp. |
| 11. | adrenal inhibitor*.mp. |
| 12. | spironolactone/ or spironolactone.mp. |
| 13. | eplerenone.mp. or eplerenone/ |
| 14. | canrenone.mp. or canrenone/ |
| 15. | finerenone.mp. or finerenone/ |
| 16. | esaxerenone.mp. or esaxerenone/ |
| 17. | 8 or 9 or 10 or 11 or 12 or 13 or 14 or 15 or 16 |
| 18. | exp adult/ |
| 19. | (adult* or aged or middle age* or geriatric or elder or old or ageing or aging or elderly).mp. |
| 20. | 18 or 19 |
| 21. | 7 and 17 and 20 |
| 22. | animal/ or animal*.mp. |
| 23. | 21 and 22 |
| 24. | 21 not 23 |

Supplementary Table 2: Risk of bias (RoB) assessments.

|  | **Domain 1: RoB arising from the randomization process** | **Domain 2: RoB due to deviations from the intended interventions** | **Domain 3: Missing outcome data** | **Domain 4: RoB in the measurement of the outcome** | **Domain 5: RoB in the selection of the reported result** | **Domain S: Bias arising from the period and carry-over effect** | **Overall RoB** |
| --- | --- | --- | --- | --- | --- | --- | --- |
| Adlin 1972 | Some concerns | Some concerns | High | Low | High | N/A | High |
| Carey 1972 | Some concerns | Some concerns | Low | Low | Some concerns | Low | Some concerns |
| Vaughan 1973 | High | Some concerns | Low | Some concerns | High | N/A | High |
| Douglas 1974 | Some concerns | Some concerns | Low | Low | Some concerns | Low | Some concerns |
| Spark 1974 | Some concerns | Some concerns | Low | Low | Some concerns | Low | Some concerns |
| Hunyor 1975 | Low | Some concerns | Low | Low | High | High | High |
| Thomas 1976 | Some concerns | Some concerns | Some concerns | Low | High | Some concerns | High |
| Brooks 1977 | Some concerns | Some concerns | Low | Low | Some concerns | Low | Some concerns |
| Ferguson 1977 | Some concerns | Some concerns | Some concerns | Low | Some concerns | Some concerns | Some concerns |
| DeCarvalho 1980 | Some concerns | Some concerns | Some concerns | Low | High | High | High |
| Kreeft 1983 | Some concerns | Some concerns | Low | Low | Some concerns | Low | Some concerns |
| Flack 2003 | Low | Low | Some concerns | Low | High | N/A | High |
| Saruta 2004 | Some concerns | Low | Some concerns | Low | Low | N/A | Some concerns |
| Williams 2004 | Some concerns | Low | Low | Low | Some concerns | N/A | Some concerns |
| Saha 2005 | Some concerns | Low | Some concerns | Low | Some concerns | N/A | Some concerns |
| Weinberger 2005 | Some concerns | Low | Low | Low | Low | N/A | Some concerns |
| Hood 2007 | Low | Low | Low | Low | Some concerns | High | High |

Supplementary Table 3. Effect of mineralocorticoid receptor antagonists and comparators on SBP, DBP and MAP.

| **Drug class** | **Study** | **Treatment** | **MRA mean change in BP (SD) mmHg** | | **Comparator mean change in BP (SD) mmHg** | | **MAP mean change (SD) mmHg** | |
| --- | --- | --- | --- | --- | --- | --- | --- | --- |
|  |  |  | **∆SBP** | **∆DBP** | **∆SBP** | **∆DBP** | **MRA** | **Comparator** |
| MRA vs diuretics | Adlin 1972 | Sp 200mg vs Hct 100mg | -24.0^§^ | -11.0^§^ | -29.0^§^ | -16.0^§^ |  |  |
|  | Vaughan 1973 | Sp 100-400mg vs Ch 50-100mg | -28.3^‡^ (17.8) | -18.1^‡^ (12.4) | -17.9^‡^ (19.7) | -16.4^‡^ (11.0) |  |  |
|  | Douglas 1974 | Sp 400mg vs Hct 100+Tr 200mg | -29.5*(15.4)  -34.9^†^(19.5) | -14.1*(6.6)  -15.9^†^(5.9) | -23.5*(17.0)  -29.3^†^(17.2) | -11.7*(7.4)  -13.1^†^(8.0) | -20.7(8.1) | -17.0(9.3) |
|  | Spark 1974 | Sp 400mg vs Hct 200mg | -32.0^\|\|^ | -25.0^\|\|^ | -13.9^\|\|^ | -8.0^\|\|^ |  |  |
|  | Hunyor 1975 | Sp 200mg, Sp 300-400mg vs Ch100mg | NR | NR | NR | NR | Sp200 -7.5%*  Sp400-14.5%* | -12%* |
|  | Brooks 1977 | Sp 50-200mg vs Hct 50-200mg | NR | NR | NR | NR | -24.6^‡^ (2.6) | 18.9^‡^ (2.9) |
|  | Ferguson 1977 | Sp 400mg vs Hct 100mg | -13.5(15.9) * ^#^  -12.6(14.3) ^†#^ | -3.9 (11.9) * ^#^  -4.6(9.6) ^†#^ | -12.6(14.3) * ^#^  -10.2(15.9) ^†#^ | -7.3(6.3) * ^#^  -6.8(11.9) ^†#^ |  |  |
|  | Kreeft 1983 | Sp 400mg vs Ch 100mg | NR | NR | NR | NR | -10.5*  -16.3^†^ | -12.1*  -15.6^†^ |
|  | Hood 2007 | Sp100mg vs Be 5mg | -11.6^‡^ (13.8) | -1.3^‡^ (9.1) | -10.5^‡^ (10.4) | -4.0^‡^ (8.2) |  |  |
| MRA vs RAAS inhibitors | Flack 2003 | Ep 50-200mg vs Lo 50-100mg | -15.8(17.5) ^‡ #^ | -12.9(7.2) ^‡ #^ | -4.9(14.9) ^‡ #^ | -6.4(7.4) ^‡ #^ |  |  |
|  | Williams 2004 | Ep 50-200mg vs En10-40mg | -15.3^‡^ | -11.1^‡^ | -10.3^‡^ | -9.0^‡^ |  |  |
|  | Weinberger 2005 | Ep 100-200mg vs Lo 50-100mg | -15.8^‡^(15.8) | -9.3^‡^(8.9) | -10.1^‡^ (15.4) | -6.7^‡^ (8.7) |  |  |
|  | Hood 2007 | Sp 100mg vs Lo 100mg | -11.6^‡^ (13.8) | -1.3^‡^ (9.1) | -5.9^‡^ (17.7) | -3.7^‡^ (10.0) |  |  |
| MRA vs ENaC inhibitors | DeCarvalho 1980 | Sp 100-mg vs Tr 100-mg | -24*  -31^†^ | -12*  -13^†^ | -9*  -15^†^ | -11*  -5^†^ |  |  |
|  | Saha 2005 | Sp 25mg vs Am 10mg | -4.6^‡^ (7.7) | -1.8^‡^ (4.8) | -9.8 (8.2) | -3.4 (5.1) |  |  |
|  | Hood 2007 | Sp 100mg vs Am 40mg | -11.6^‡^ (13.8) | -1.3^‡^ (9.1) | -11.5^‡^ (13.7) | -3.1^‡^ (10.0) |  |  |
| MRA vs placebo | Carey 1972 | Sp 400mg compared to placebo | -32.0*(17.3)  -34.8^†^(17.9) | -14.6*(9.9)  -17.0^†^(10.3) | NR | NR | -20.5*(11.7)  -22.9^†^(12.2) |  |
|  | Saruta 2004 | Ep 50,100,200mg and placebo | -6.8^‡^ -9.7^‡^ -10.6^‡^ | - 5.1^‡^ -6.9^‡^ -7.5^‡^ | -2.1^‡^ | -3.0^‡^ |  |  |
| MRA vs β blocker | Thomas 1976 | Sp 200-400mg vs Ox 160-640mg | -45.2^\|\|^ (23.1) | -23.9^\|\|^ (7.2) | -31.7^\|\|^ (16.8) | -20.8^\|\|^ (11.2) |  |  |
| MRA vs α-2 agonist | Thomas 1976 | Sp 200-400mg vs Me 750-3000mg | -45.2^\|\|^ (23.1) | -23.9^\|\|^ (7.2) | -32.2^\|\|^ (14.9) | -23.1^\|\|^ (7.9) |  |  |

*supine; ^†^erect; ^‡^seated; ^§^average of supine and erect; ^||^not specified; ^#^derived from graph. Am: amiloride; α: alpha; Be: Bendroflumethiazide; β: beta; BP: blood pressure; Ch: chlorthalidone; DBP: diastolic blood pressure; ENaC: epithelial sodium channel; En: enalapril; Ep: eplerenone; Hct: hydrochlorothiazide; Lo: losartan; MAP: mean arterial pressure; Me: methyldopa; MRA: mineralocorticoid receptor antagonist; NR: not reported; Ox: oxprenolol; SBP: systolic blood pressure; Sp: spironolactone; SD: standard deviation; Tr: triamterene.

Supplementary Table 4: Sensitivity analysis of meta-analysis with study omission (mineralocorticoid receptor antagonists versus diuretics).

|  |  | **SBP** | | | **DBP** | | |
| --- | --- | --- | --- | --- | --- | --- | --- |
| **Study omitted** | | **Mean difference* mmHg [95%CI]** | **p-value** | **I^2^ %** | **Mean difference* mmHg [95%CI]** | **p-value** | **I^2^ %** |
| Individual | Adlin 1972 | -6.9 [-15.0, 1.2] | 0.09 | 63 | -2.5 [-10.1, 5.0] | 0.51 | 79 |
|  | Vaughan 1973 | -3.6 [-11.9, 4.7] | 0.39 | 65 | -0.8 [-8.4, 6.7] | 0.83 | 80 |
|  | Spark 1974 | -1.5 [-5.8, 2.8] | 0.50 | 6 | 2.5 [-0.2, 5.2] | 0.07 | 0 |
|  | Ferguson 1977 | -5.6 [-14.5, 3.3] | 0.22 | 70 | -1.8 [-9.3, 5.6] | 0.63 | 81 |
|  | Hood 2007 | -6.3 [-16.4, 3.7] | 0.22 | 64 | -2.3 [-11.0, 6.4] | 0.61 | 78 |
| High Risk of bias | Adlin, Vaughan and Hood | -10.1 [-26.1, 6.0] | 0.22 | 71 | -6.9 [-26.3, 12.5] | 0.48 | 89 |

*Aggregated mean treatment group difference was calculated by mean change in blood pressure with mineralocorticoid receptor antagonists minus the mean change in blood pressure with comparator.

DBP: diastolic blood pressure; SBP: systolic blood pressure.

Supplementary Table 5: Sensitivity analysis of meta-analysis using correlation coefficient derived from Douglas *et al* to impute change in blood pressure standard deviation for Spark *et al* (mineralocorticoid receptor antagonists versus diuretics).

|  | **SBP** | | | **DBP** | | |
| --- | --- | --- | --- | --- | --- | --- |
|  | **Mean difference* mmHg [95%CI]** | **p-value** | **I^2^ %** | **Mean difference* mmHg [95%CI]** | **p-value** | **I^2^ %** |
| Douglas et al | -4.8 [-11.9, 2.4] | 0.19 | 60 | -1.0 [-5.7, 3.6] | 0.66 | 72 |
| Plus 0.2 | -5.3 [-13.0, 2.5] | 0.18 | 69 | -1.4 [-8.4, 5.5] | 0.68 | 81 |
| Minus 0.2 | -4.3 [-11.1, 2.5] | 0.21 | 53 | -0.5 [-6.1, 5.2] | 0.87 | 67 |

*Aggregated mean treatment group difference was calculated by mean change in blood pressure with mineralocorticoid receptor antagonists minus the mean change in blood pressure with comparator.

DBP: diastolic blood pressure; SBP: systolic blood pressure.

Supplementary Table 6: Sensitivity analysis of meta-analysis with study omission (mineralocorticoid receptor antagonists and versus angiotensin-converting enzyme inhibitors/angiotensin receptor blockers)

|  |  | **SBP** | | | **DBP** | | |
| --- | --- | --- | --- | --- | --- | --- | --- |
| **Study omitted** | | **Mean difference* mmHg [95%CI]** | **p-value** | **I^2^ %** | **Mean difference* mmHg [95%CI]** | **p-value** | **I^2^ %** |
| All | Flack 2003 | -5.5 [-8.5, -2.4] | <0.01 | 0 | -1.0 [-3.8, 1.7] | 0.46 | 60 |
|  | Williams 2004 | -7.5 [-11.0, -4.0] | <0.01 | 22 | -2.5 [-7.4, 2.4] | 0.32 | 88 |
|  | Weinberger 2005 | -7.3 [-11.1, -3.5] | <0.01 | 32 | -2.3 [-7.3, 2.7] | 0.36 | 88 |
|  | Hood 2007 | -7.1 [-10.7, -3.5] | <0.01 | 35 | -3.9 [-6.9, -0.9] | 0.01 | 74 |
| High RoB | Flack and Hood | -5.4 [-8.8, -1.9] | <0.01 | 0 | -2.4 [-4.3, -0.4] | 0.02 | 0 |

*Aggregated mean treatment group difference was calculated by mean change in blood pressure with mineralocorticoid receptor antagonists minus the mean change in blood pressure with comparator.

DBP: diastolic blood pressure; SBP: systolic blood pressure.

Supplementary Table 7: Sensitivity analysis of meta-analysis with study omission (mineralocorticoid receptor antagonists versus epithelial sodium channel inhibitors)

|  |  | **SBP** | | | **DBP** | | |
| --- | --- | --- | --- | --- | --- | --- | --- |
| **Study omitted** | | **Mean difference* mmHg [95%CI]** | **p-value** | **I^2^ %** | **Mean difference* mmHg [95%CI]** | **p-value** | **I^2^ %** |
| All | DeCarvalho 1980 | 2.8 [-2.4, 7.9] | 0.30 | 55 | 1.67 [-0.6, 3.9] | 0.14 | 0 |
|  | Saha 2005 | -6.4 [-21.3, 8.4] | 0.40 | 76 | 1.16 [-2.6, 4.9] | 0.54 | 2 |
|  | Hood 2007 | -4.1 [-24.2, 16.1] | 0.69 | 87 | 1.27 [-1.4, 4.0] | 0.36 | 0 |
| High RoB | DeCarvalho and Hood | 5.2 [0.8, 9.7] | 0.02 | NA | 1.6 [-1.2, 4.4] | 0.26 | NA |

*Aggregated mean treatment group difference was calculated by mean change in blood pressure with mineralocorticoid receptor antagonists minus the mean change in blood pressure with comparator.

DBP: diastolic blood pressure; SBP: systolic blood pressure.

Supplementary Table 8: Sensitivity analysis of meta-analysis using correlation coefficient derived from Douglas et al to impute change in blood pressure standard deviation for DeCarvalho et al (mineralocorticoid receptor antagonists versus epithelial sodium channel inhibitors).

|  | **SBP** | | | **DBP** | | |
| --- | --- | --- | --- | --- | --- | --- |
|  | **Mean difference* mmHg [95%CI]** | **p-value** | **I^2^ %** | **Mean difference* mmHg [95%CI]** | **p-value** | **I^2^ %** |
| Douglas et al | -0.9 [-9.0, 7.1] | 0.82 | 77 | 1.5 [-0.7, 3.6] | 0.19 | 0 |
| Plus 0.2 | -2.0 [-10.9, 6.9] | 0.66 | 83 | 1.3 [-0.8, 3.5] | 0.22 | 0 |
| Minus 0.2 | -0.1 [-7.6, 7.4] | 0.98 | 72 | 1.5 [-0.7, 3.7] | 0.18 | 0 |

*Aggregated mean treatment group difference was calculated by mean change in blood pressure with mineralocorticoid receptor antagonists minus the mean change in blood pressure with comparator.

DBP: diastolic blood pressure; SBP: systolic blood pressure.
